# Yeast Killer Toxin-Like Candidacidal Ab6 Antibodies Elicited through the Manipulation of the Idiotypic Cascade

**DOI:** 10.1371/journal.pone.0105727

**Published:** 2014-08-27

**Authors:** Luciano Polonelli, Concetta Beninati, Giuseppe Teti, Franco Felici, Tecla Ciociola, Laura Giovati, Martina Sperindè, Carla Lo Passo, Ida Pernice, Maria Domina, Milena Arigò, Salvatore Papasergi, Giuseppe Mancuso, Stefania Conti, Walter Magliani

**Affiliations:** 1 Dipartimento di Scienze Biomediche, Biotecnologiche e Traslazionali, Unità di Microbiologia e Virologia, Università degli Studi di Parma, Parma, Italy; 2 Metchnikoff Laboratory, Dipartimento di Scienze Pediatriche, Ginecologiche, Microbiologiche e Biomediche, Università degli Studi di Messina, Messina, Italy; 3 Dipartimento di Bioscienze e Territorio (DiBT), Università degli Studi del Molise, Contrada Fonte Lappone, Pesche (IS), Italy; 4 Dipartimento di Scienze Biologiche ed Ambientali, Università degli Studi di Messina, Messina, Italy; University of Wisconsin, Food Research Institute, United States of America

## Abstract

A mouse anti-anti-anti-idiotypic (Id) IgM monoclonal antibody (mAb K20, Ab4), functionally mimicking a *Wyckerhamomyces anomalus* (*Pichia anomala*) killer toxin (KT) characterized by fungicidal activity against yeasts presenting specific cell wall receptors (KTR) mainly constituted by β-1,3-glucan, was produced from animals presenting anti-KT Abs (Ab3) following immunization with a rat IgM anti-Id KT-like mAb (mAb K10, Ab2). MAb K10 was produced by immunization with a KT-neutralizing mAb (mAb KT4, Ab1) bearing the internal image of KTR. MAb K20, likewise mAb K10, proved to be fungicidal *in vitro* against KT-sensitive *Candida albicans* cells, an activity neutralized by mAb KT4, and was capable of binding to β-1,3-glucan. MAb K20 and mAb K10 competed with each other and with KT for binding to *C. albicans* KTR. MAb K20 was used to identify peptide mimics of KTR by the selection of phage clones from random peptide phage display libraries. Using this strategy, four peptides (TK 1-4) were selected and used as immunogen in mice in the form of either keyhole limpet hemocyanin (KLH) conjugates or peptide-encoding minigenes. Peptide and DNA immunization could induce serum Abs characterized by candidacidal activity, which was inhibited by laminarin, a soluble β-1,3-glucan, but not by pustulan, a β-1,6-glucan. These findings show that the idiotypic cascade can not only overcome the barrier of animal species but also the nature of immunogens and the type of technology adopted.

## Introduction

The concept of idiotypy could be dated back to the beginning of the XX century when Paul Ehrlich and others predicted that antibodies (Abs) may be directed against the combining regions of other Abs. At that time, nothing was known about the molecular properties of Abs and the vague term “side chain” was used to define particular chemical structures of the combining site (afterwards referred to as idiotype, Id) which could account for differences in its specificity [1]. Ehrlich already visualized the possibility that side chains of Abs might resemble the three dimensional structure of the antigen (Ag), thus anticipating Jerne's late theory of “internal image” [2].

The real era of research on idiotypy started with the work of Oudin and Michel [3], and Kunkel et al. [4], who described anti-Id Abs as markers distinguishing the variable regions of specific Ab molecules. Experimental and clinical studies have shown that animals and humans are capable of producing anti-Ids to their own immunoglobulins (Igs) [5], [6]. Four categories of anti-Id have been identified (Ab2α, Ab2β, Ab2γ, Ab2ε) and the most intriguing are Ab2β, which are complementary to the Ab1 paratope and represent the internal image of the Ag, leading to the proposal of using Ab2β anti-Ids as surrogate vaccines [7]–[11]. One of the criteria for structural similarity of epitopes on the Ag and anti-Ids is the ability of anti-Ids to induce the synthesis of anti-anti-Ids (Ab3) recognizing the external Ag [12]. There have been numerous reports on the interaction of anti-Ids with cellular receptors for a variety of external Ags [13]. The interaction with cellular receptors, especially if the appropriate biological effects are mediated, is perhaps more convincing than the induction of Abs as evidence for the structural relatedness of Ag and anti-Id.

In a previous work we described a rat anti-Id mAb (mAb K10, Ab2) representing the internal image of a killer toxin (KT, Ag), produced by the yeast *Wyckerhamomyces anomalus* (*Pichia anomala*) ATCC 96603, characterized by fungicidal activity against *Candida albicans* cells bearing specific KT cell wall receptors (KTR) [14]. MAb K10 was produced by immunization of animals with a KT-neutralizing mAb (mAb KT4, Ab1) which proved to have functional relatedness to KTR [15]–[19]. MAb K10 competed with KT for binding to specific KTR, distributed mainly in budding cells and germination tubes, which consist essentially of β-1,3-glucans [16], [18], [19]. MAb KT4 was able indeed to neutralize the candidacidal activity of mAb K10 against KT-sensitive *C. albicans* cells [14].

This work deals with the production and functional characterization of the KT-like anti-anti-anti-Id mAb K20 (Ab4), which occurs in the course of the idiotypic cascade following immunization with mAb K10 (Ab2), and its potential to select peptide mimics of KTR from random peptide phage display libraries able to elicit candidacidal Abs (Ab6).

## Materials and Methods

### Ethics statement

The *in vivo* experiments were performed at the animal facilities of the Universities of Parma and Messina according to the European guidelines for handling of laboratory animals. Protocols were approved by the local committees on the ethics of animal experiments (Comitato etico per la sperimentazione animale of the University of Parma, Permit Number: 40/07, and Comitato etico per la sperimentazione animale of the University of Messina, Permit Number: 04052007). All efforts were made to minimize pain and suffering.

### Yeast isolates and KT production

*Wyckerhamomyces anomalus* (*P. anomala*) ATCC 96603 was used for KT production according to a previously described procedure [15]. The 50× concentrated KT, to be used as reference antigen in ELISA, was tested for killer activity by a conventional well assay against *C. albicans* UP10S, employed as reference KT-susceptible strain throughout the study [20].

### Immunization of mice, detection of polyclonal Ab3, and production of hybridomas

KT-like mAb K10, used as immunogen in this study, is a rat IgM which proved to exert a candidacidal activity *in vitro* and a therapeutic effect in an experimental model of vaginal candidiasis [14], [21]. Two Balb/C female mice (5 week old, 18 g body weight) were immunized twice subcutaneously with 50 µg of purified mAb K10 in 50 µl of complete Freund's adjuvant (day 0), or incomplete FA (day 15). The animals were then injected intraperitoneally (i.p.) with the same amount of immunogen in saline at days 21 and 35.

A conventional ELISA using KT as coating Ag was performed to verify the presence of Ab3 polyclonal Abs in the serum of mAb K10-immunized animals collected at days 0 (before immunization), 21 and 42 (1 week after the fourth Ag injection). 96 well microtiter plates were coated with 100 µl of 50× KT diluted 1∶25 in carbonate buffer, pH 9.6, by overnight incubation at 37°C. The plates were then washed with phosphate buffered saline (PBS) containing 0.05% Tween 20 (PBS-T) and blocked (1 h, 37°C) with 1% bovine serum albumin (BSA) in PBS 0.15 M, pH 7.3. After washing, 100 µl of mouse serum diluted 1∶200 in PBS-BSA were distributed in each well, and the plates were incubated for 2 h at room temperature (RT). After further washing, the bound Abs were revealed by adding (100 µl/well) horseradish peroxidase (HRP)-conjugated goat anti-mouse Ig, diluted 1∶5000 in PBS-BSA (1 h, RT). After repeated washing, 100 µl/well of a tetramethylbenzidine dihydrochloride solution in citrate buffer and H_2_O_2_ were added, and the plates incubated in the dark for 20 min. The reaction was stopped with the addition of 50 µl/well of 10 M solution of sulfuric acid and the absorbance was measured at 450 nm by a micro-plate reader. Tests were done in duplicate.

A booster injection of mAb K10 was performed i.p. in mice three days before fusion. A mouse was then sacrificed and its spleen cells fused with NS0 myeloma cells as previously described [15].

The supernatant of each hybridoma produced in the fusion was screened for antifungal activity against *C. albicans* UP10S. The supernatants (200 µl) were transferred to the wells of a microtiter plate and then 10 µl of a *C. albicans* cell suspension (1×10^5^ cells/ml) were added to each well. The controls were wells in which the supernatant of the myeloma cells was utilized in place of the hybridoma's supernatant. After incubation for 24 h at 30°C, the microtiter plates were read for optical density with a spectrophotometer at 550 nm. The hybridoma, whose supernatant produced the highest inhibition of the yeast's growth in the screening assay, was expanded and cloned. The mAb produced by the clone, whose supernatant showed the highest candidacidal activity, was designated as mAb K20 and further characterized.

### Determination of mAb K20 isotype

The Ig class determination of mAb K20 was performed by immunoenzymatic assay using rat mAbs directed against the H chain of mouse Ig (μ, γ1, γ2a, γ2b, and γ3) (University of Louvain, Belgium). In particular, couples of each isotype-specific Ab were used, a capture mAb for coating and a HRP-conjugated mAb for detection. The same reagents were used in ELISA for mAb quantification.

### Determination of mAb K20 specificity

A direct ELISA assay was performed to evaluate the binding of mAb K20 to mAb KT4 by using mAb KT4-coated microtiter plates and purified mAb labeled with biotin. Biotin-labeling was carried out according to previously described procedures [14]. The bound Ab was revealed by adding (100 µl/well) HRP-conjugated streptavidin diluted 1∶5000 in PBS-BSA (1 h, RT) and, then, the proper substrate as previously described. The same assay was also carried out adding to the biotinylated mAb increasing amounts of the same unlabeled mAb, mAb K10, or an isotype-matched unrelated mAb.

### Immunofluorescence studies

Immunofluorescence studies were carried out by using mAb K20 labeled with biotin. *C. albicans* UP10S cells were suspended in 199 medium and incubated at 37°C for 3 h under shaking conditions. The yeast suspension was centrifuged (900 *g*, 10 min) and the cell pellet was washed twice with sterile distilled water. The pellet was then suspended in PBS and the suspension adjusted to give a final concentration of approximately 50 cells/field at 40× magnification. A standardized yeast cell suspension (20 µl) was put into each well of an immunofluorescence slide and, after drying, fixed by gentle heating. Biotinylated mAb diluted 1∶100 in PBS (20 µl) was added to each well and maintained for 1 h at 37°C in a humid chamber. The slide was successively washed three times in PBS (10 min each) with shaking and allowed to dry at RT; 20 µl of streptavidin-fluorescein diluted 1∶100 in Evans blue were then added to each well and allowed to react for 20 min under the same conditions as above. The slides were then rinsed with distilled water, mounted with a cover slip using a mounting fluid, and observed under a fluorescence microscope (Nikon Optiphot, Tokyo). As a negative control, the immunofluorescence assay was carried out by using, in the same procedure, PBS in place of biotinylated mAb. In further experiments, biotinylated mAb was mixed with mAb KT4 or unlabeled mAb, mAb K10, or an isotype-matched mAb, at various concentrations, before being added to the wells of the immunofluorescence slide.

### Selection of KTR-like peptides from random peptide libraries

To select peptides mimicking KTR, mAb K20 was used to screen four previously generated random peptide libraries of 9 or 12 amino acids, named pVIII-9aa, pVIII-9aa.Cys, pVIII.Cys-Cys and pVIII-12aa [22], [23]. These libraries carry random inserts encoding peptides fused to the N-terminal region of pVIII, the major coat protein of the M13 filamentous phage. The pVIII-9aa.Cys and pVIII.Cys-Cys libraries respectively display 9 or 12-amino acid inserts containing two cysteine residues to cyclically constrain the peptides. Different pools of libraries, (pVIII-12aa/pVIII.Cys-Cys or pVIII-9aa.Cys/pVIII.Cys-Cys or pVIII-9aa/pVIII-9aa.Cys) were used to select phages as previously described [24]. After a total of three rounds of affinity selection, the positive phage clones were identified through plaque immunoscreening and enzyme immunoassay, as previously described [25], using plates coated with rat anti-coat protein III (pIII) mAb (1 µg/ml) followed by addition of purified supernatant phage (10^10^ pfu/ml) and by mAb K20 or an isotype-matched mAb (1 µg/ml). Binding was detected with alkaline phosphatase-conjugated goat anti-mouse IgM Ab (1∶5000) using *p*-nitrophenyl phosphate as substrate. All ELISA-positive clones were sequenced using the dideoxy-mediated chain termination method, Cy5-50 primers and an automatic sequencer (ALFexpress, Amersham).

### DNA constructs

To generate plasmids for DNA vaccination, a previously described plasmid expression vector designated as pT.neo was used [26]. The antigenic peptide sequences were inserted at the 3′ *EcoRI/XbaI* flanking site of the T helper epitope of pT.neo. For co-immunization studies, a previously described plasmid encoding for murine interferon-γ (pmIFN-γ) was used [27]. The plasmids were produced by transforming DH5a *Escherichia coli* cells and purified, after sequence analysis, using EndoFree Plasmid Maxi or Giga kits (Qiagen). Each lot of plasmid DNA had a A_260_/A_280_ ratio ≥1.8, as determined by UV spectrophotometry, endotoxin content ≤0.1 EU/µg DNA, as determined by *Limulus* Amebocyte Lysate test (Associates of Cape Cod) and a predominantly supercoiled form.

For comparison, two selected peptides were conjugated to keyhole limpet haemocyanin (KLH) to increase their antigenicity and immunogenicity.

### DNA Immunization

BALB/c mice (5-7-week old, Charles River; 5/group) were immunized with 100 µg of purified DNA (pT.TK1, pT.TK2, pT.TK3, and pT.TK4 plasmids or empty vector pT.neo) in a total volume of 50 µl of PBS. Mice were injected intramuscularly (i.m.) on day 0, 21 and 42 and tail vein bled on day 56. In the co-immunization studies, 70 µg of pmIFN-γ were mixed with the immunizing plasmid and injected in a total volume of 50 µl. Sera of the immunized animals were evaluated for *in vitro* candidacidal activity.

For comparison, groups of animals were immunized three times with KLH-conjugated peptides (40 µg) using Freund's adjuvant.

### Evaluation of candidacidal activity

The *in vitro* candidacidal activity of the selected mAb and sera from immunized animals against *C. albicans* UP10S were carried out by conventional colony forming unit (CFU) assays [20]. Briefly, 10 µl of H_2_O containing ∼2–3×10^2^ germinating yeast cells (expected number of CFU in the controls) were added to 90 µl of H_2_O in the presence or absence (control growth) of the purified mAb (final concentration 100 µg) or different dilutions of sera from immunized animals. The specificity of the killing activity was ascertained by adding the same amount of yeast cells to mAb and sera pre-incubated with mAb KT4 or with increasing amounts (from 0.2 to 100 µg/ml) of laminarin, a soluble β-1,3-glucan, pustulan, a soluble β-1,6-glucan, or the respective KLH-peptide conjugates. After 6 h of incubation at 37°C with the respective reagents, the yeast cells were dispensed and streaked on the surface of Sabouraud dextrose agar plates which were then incubated at 30°C, and CFU were enumerated after 48 h. Each experiment was carried out in triplicate for statistical purposes. The killing was expressed as percentage of CFU, calculated as: (average number of CFU in the test group/average number of CFU in the control group) ×100.

## Results

### Production of Ab4 KT-like mAb

**Figure 1:**
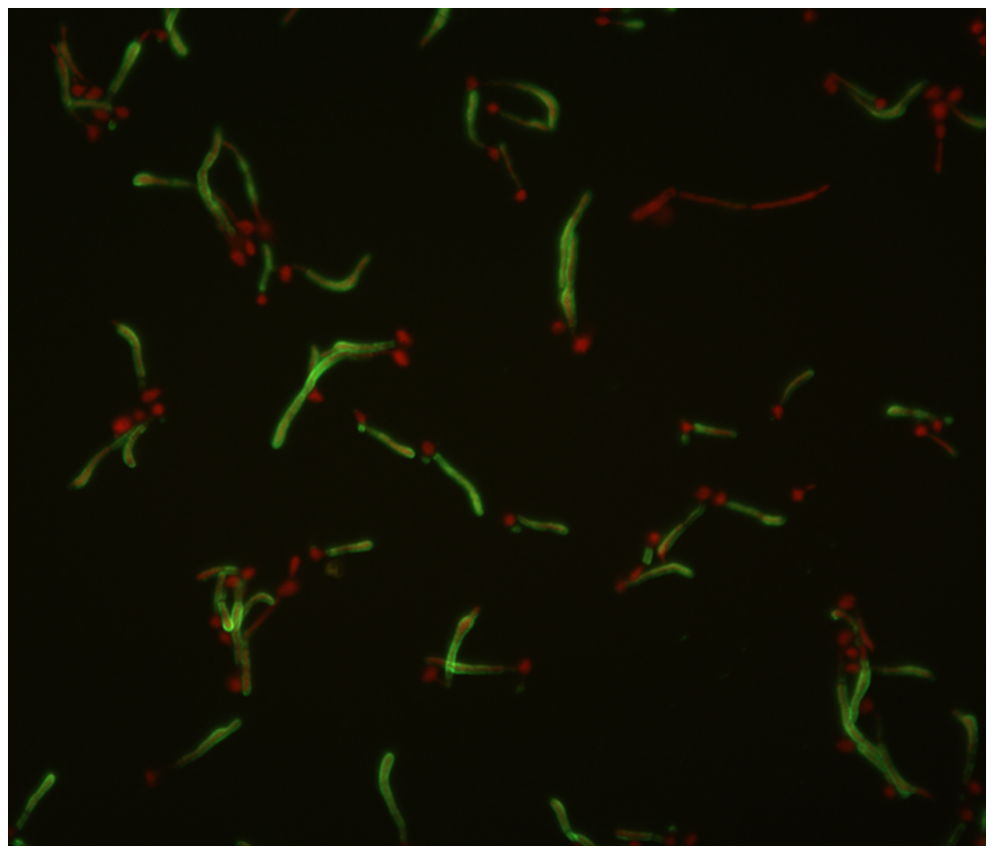
Binding of mAb K20 to *Candida albicans* cell wall, particularly on germ tubes and budding cells. MAb K20 labeled with biotin was used in the immunofluorescence assay with streptavidin-fluorescein.

Immunization of mice with KT-like mAb K10 (Ab2) elicited the production of serum Abs (Ab3) which proved to be reactive in ELISA with KT (mean O.D. immune sera 0.612, vs preimmune sera 0.145). From the spleen of a mAb K10-immunized animal it was possible to produce a hybridoma secreting an IgM mAb, named mAb K20 (Ab4), which proved to specifically react in ELISA with mAb KT4. In immunofluorescence assays, mAb K20 proved to bind to *C. albicans* cell wall, particularly to germ tubes and budding cells (Figure 1). The immunofluorescence reactivity was significantly abolished by mixing biotin-labeled mAb K20 with mAb KT4. Unlabeled mAb K20 and mAb K10 competed with labeled mAb K20 for binding to *C. albicans* cells, as their presence at the highest concentration tested caused a complete disappearance of cellular staining. At the same concentration of the unlabeled antibodies, mAb K10 proved to be more effective than mAb K20 in decreasing immunofluorescence reactivity. On the contrary, immunofluorescence did not change when an isotype-matched mAb was added to labeled mAb K20 (data not shown).

Likewise KT and mAb K10, mAb K20 proved to be killing *in vitro C. albicans* UP10S cells (99,4±0,11% inhibition in the CFU assay). The candidacidal activity of mAb K20 was neutralized by mAb KT4, like that of mAb K10 and KT.

### Selection of KTR-like peptides from random peptide libraries

**Figure 2:**
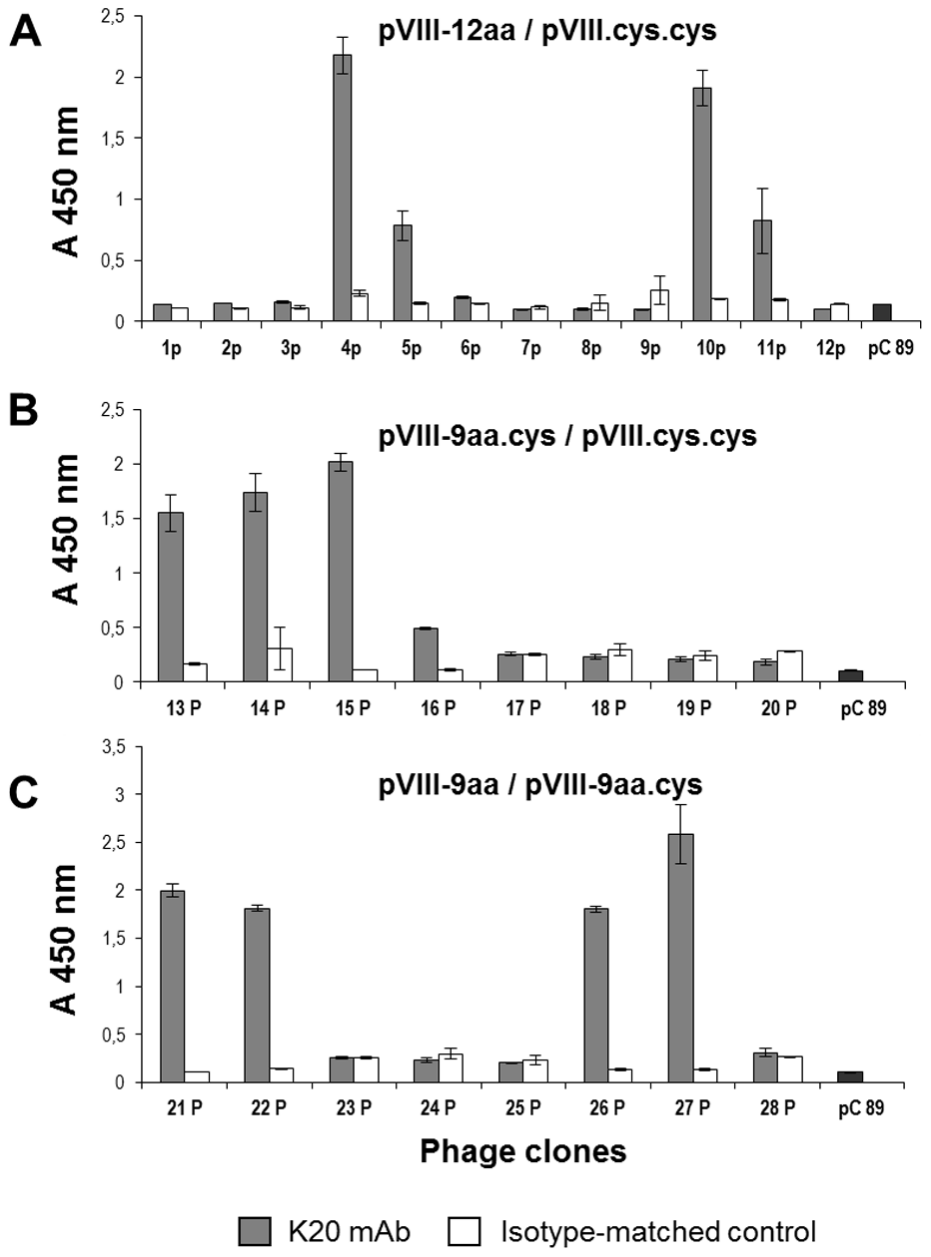
Binding of phage clones to mAb K20. Plates were sensitized with a mAb directed against phage protein III (1 µg/ml) and 100 µl of purified phage clones (10^11^ pfu/ml) were added. After 1 h incubation, mAb K20 or an isotype-matched IgM (1 µg/ml) were added. Binding was detected by using alkaline phosphatase-conjugated anti-mouse IgM Ab. Pools of libraries, (pVIII-12aa/pVIII.Cys-Cys or pVIII-9aa.Cys/pVIII.Cys-Cys or pVIII-9aa/pVIII-9aa.Cys) were used in different phage selections. Results are reported in separate panels (A, B or C). Phage pC89, displaying wild-Type pVIII, was used as negative control. Data represents the means ± the SD of three determinations.

MAb K20 was used as a template to select KTR-mimicking peptides from phage display libraries expressing random peptides. Pools, each made of two different libraries, were independently used to select mAb K20-binding phages. After three rounds of selection using mAb K20 and protein G conjugated-beads, 28 phage clones were obtained, and individually assayed for binding to mAb K20 sensitized plates using an optimized phage-ELISA assay. Twelve phage clones strongly reacted against mAb K20, but not against the isotype-matched control (Figure 2). Moreover, the specificity of these interactions was confirmed by inhibition of binding of each of these clones to mAb K20 in the presence of mAb KT4 (data not shown).

Next, the sequences of the clone inserts were determined (Table 1). Among the 12 phage clones selected, four different amino acid sequences were present and were designated TK1, TK2, TK3 and TK4. TK2 and TK3 share the consensus sequence DCHPQG, while no other sequence similarities could be discerned.

**Table 1:** Aminoacid sequences of phage inserts from mAb K20-binding clones.

Clone	Designation	Library*	Peptide sequence
4P	TK1	pVIII-12aa	IYNFINQAWRHA
5P	TK2	pVIII.Cys-Cys	DCHPQGDRFCSQ
10P		pVIII.Cys-Cys	DCHPQGDRFCSQ
11P		pVIII.Cys-Cys	DCHPQGDRFCSQ
15P		pVIII.Cys-Cys	DCHPQGDRFCSQ
13P	TK3	pVIII.Cys-Cys	DCHPQGGRVCFH
14P		pVIII.Cys-Cys	DCHPQGGRVCFH
16P		pVIII.Cys-Cys	DCHPQGGRVCFH
21P	TK4	pVIII-9aa	GCVHNICFA
22P		pVIII-9aa	GCVHNICFA
26P		pVIII-9aa	GCVHNICFA
27P		pVIII-9aa	GCVHNICFA

*peptide libraries of 12 (pVIII-12aa and pVIII.Cys-Cys) or 9 (pVIII-9aa) amino acids.

### Immunogenicity of the selected peptides

**Figure 3:**
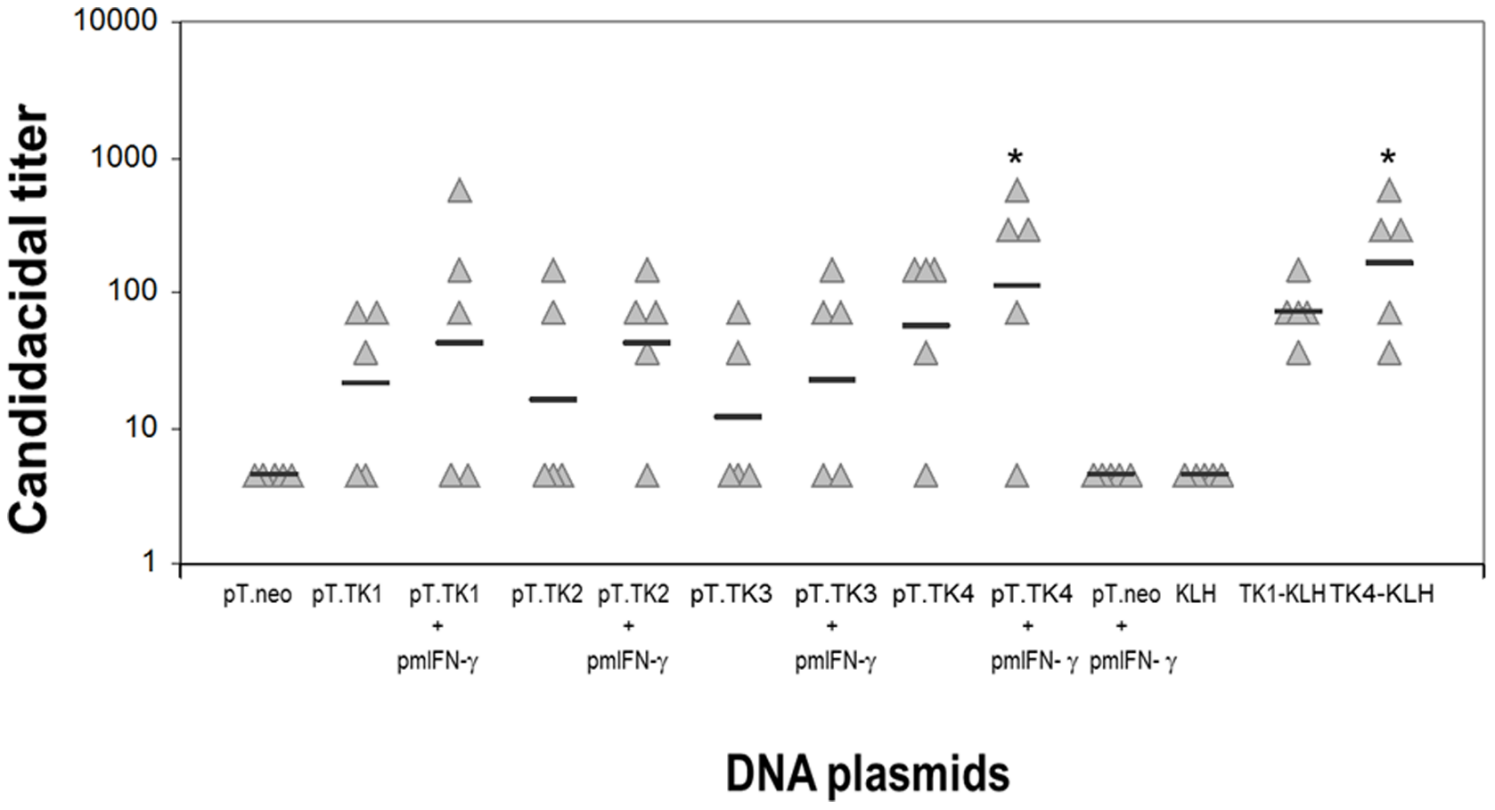
Candidacidal titers in immunized animals. Hundred µg of plasmids containing the selected peptide sequences (pT.TK1, pT.TK2, pT.TK3, and pT.TK4) or empty vector (pT.neo) were given three times i.m. and serum was collected at day 56 after the first immunization. To verify candidacidal titer elevation, the plasmids above described were also co-administered with a plasmid encoding for murine interferon-γ (pmIFN-γ) (70 µg). For comparison, groups of animals were immunized three times with the TK1 and TK4 peptide conjugated to keyhole limpet hemocyanin (KLH) (40 µg) using Freund's adjuvant. To calculate mean geometric titers (horizontal bars), sera without detectable candidacidal activity were given an arbitrary titer of 4.5 (i.e. half of the reciprocal of the lowest dilution tested). *, *P*<0.05 =  significantly different from pT.neo or KLH alone by ANOVA and Student-Newman-Keuls test.

In initial studies, the immunogenicity of the TK 1–4 peptides was explored, using a previously optimized DNA immunization strategy, which allowed to quickly screen these constructs. Oligodeoxynucleotides encoding for TK1, TK2, TK3 and TK4 peptides were optimized for codon usage and cloned in the mammalian vector pT.neo suitable for DNA vaccination. All constructs, which were named pT.TK1, pT.TK2, pT.TK3 and pT.TK4, encoded for a T helper epitope of the C fragment of the tetanus toxin, fused to the 5′ of the peptide-encoding sequence. The composition of the immunizing plasmids is summarized in Table 2. Of note, none of these constructs contained leader sequences, which are sometimes used to increase the extracellular delivery of antigens in DNA immunization strategies. Sera from mice immunized i.m. three times with the different plasmids (100 µg each in 50 µl of PBS), at 21 day-intervals, were collected two weeks after the last immunization and assayed for their ability to kill *C. albicans in vitro*. Results are expressed in terms of candidacidal titers, i.e. the inverse of the highest serum dilution that still caused a 50% reduction in CFU (Figure 3). A proportion of the TK 1–4 gene-immunized animals developed candidacidal serum activity, which was not detected in animals administered with the empty vector (pT-neo, Figure 3) or in preimmune serum samples (data not shown). In particular, responding animals were 4/5 in the pT.TK4-immunized group, and 3, 2 and 2 of 5 mice immunized with pT.TK1, pT.TK2, and pT.TK3, respectively. Co-administration of plasmids containing the IFN-γ gene increased the number of responding animals (from 2 to 4 for pT.TK2-immunized group and from 2 to 3 for pT.TK2-immunized group) or the candidacidal titer (pT.TK1 and pT.TK4-immunized groups), while administration of pmIFN-γ alone did not induce candidacidal Abs (Figure 3). When the ability of peptide-protein conjugates to induce candidacidal Abs was assessed, the TK4 peptide conjugated to KLH (TK4-KLH) was found to induce significant candidacidal activity (Figure 3).

**Table 2:** Plasmids used for immunization studies.

Description	Antigenic sequence
pT.neo*	MKLQYIKANSKFIGITELEF
pT.TK1	MKLQYIKANSKFIGITELEF**IYNFINQAWRHA**GDPAK
pT.TK2	MKLQYIKANSKFIGITELEF**DCHPQGDRFCSQ**PDPAK
pT.TK3	MKLQYIKANSKFIGITELEF**DCHPQGGRVCFH**PDPAK
pT.TK4	MKLQYIKANSKFIGITELEF**GCVHNICFA**DPAK
pmIFN-γ**	Murine IFN-γ

*pT.neo is a previously described plasmid espression vector [26] used for insertion of the selected peptide sequences (plasmids pT.TK1–4),

**pmIFN-γ is a previously described plasmid encoding for murine interferon-γ [27] used for co-immunization studies.

Letters in bold indicate the antigenic peptides selected from phage displayed libraries

**Figure 4:**
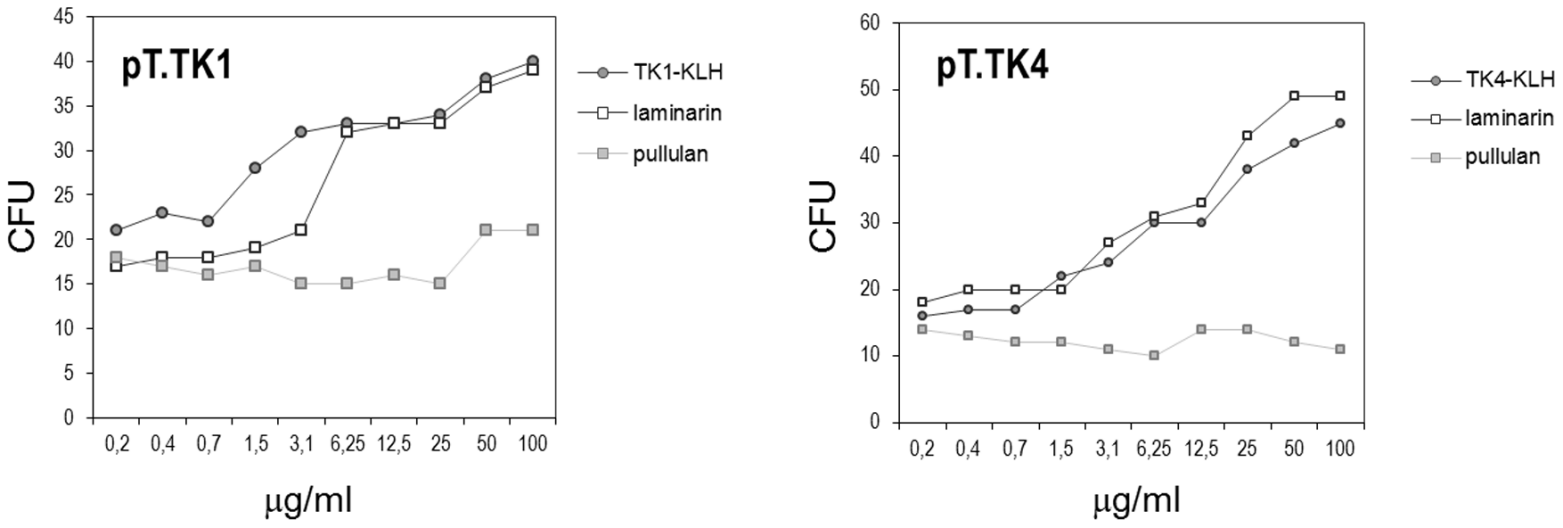
Inhibition of the candidacidal activity of sera from animals immunized with plasmids containing selected peptide sequences (pT.TK1, left panel and pT.TK4, right panel) by laminarin, a soluble β-1,3-glucan, pustulan, a soluble β-1,6-D-glucan, and keyhole limpet hemocyanin (KLH)-conjugated peptides.

To verify that the peptide-induced serum Abs were directed against their intended target, i.e. the β-1,3-glucan KTR, inhibition studies were performed on selected sera from the experiments detailed in Figure 3. Figure 4 shows that the candidacidal activity of these sera was inhibited by laminarin, a soluble β-1,3-glucan, but not by pustulan, a soluble β-1,6-D-glucan. Moreover, candidacidal activity was inhibited by the respective KLH-conjugated peptides (Figure 4).

## Discussion

According to the idiotypic network theory of Jerne [2] an Ag can elicit Abs (Ab1) which express an idiotype (Id) against which an anti-Id response (Ab2) can be induced, which in turn may induce an anti-anti-Id response (Ab3). Since external Ag and Ab2 can bind competitively to the same region of an Ab1, some Ab2 can carry the “internal image” of the external Ag and therefore can induce immunity to it. The criteria for the definition of structural identity between Ag and anti-Id Ab imply the capacity of anti-Id Ab to elicit the production of anti-Ag Abs, to interact with Ag specific receptors, to compete with Ag for the binding site of anti-Ag Ab, to circumvent the restriction of MHC barrier and to mimic the intrinsic biological activity of Ag, if any [12].

In this study we demonstrated that mAb K20 (Ab4), obtained in mice by immunization with the rat mAb K10 (Ab2) through the production of Ab3 reacting with KT (Ag), does represent the functional internal image of the Ag, being characterized by similar candidacidal activity.

As described in Table 3, overall, our results furtherly support the Jerne's theory and prove that mAb K20 acts as the internal image of KT, as well as peptides that bind mAb K20 act as functional KTR mimics. The relevance of these findings resides in the consideration that mAbs sharing common Ids (such as mAb K10 and mAb K20) could be produced after immunization with Ags represented by different mAbs (such as mAb KT4 and mAb K10 itself). From independent fusion experiments two hybridoma cell lines secreting KT-like mAbs, one representing an anti-Id (mAb K10) and another an anti-anti-anti-Id (mAb K20), which exhibited similar anti-mAb KT4 and anti-*Candida* activity, were obtained in two animal species.

**Table 3:** Schematic representation of killer toxin (KT)/killer toxin receptor (KTR) functional idiotypic cascade.

Definition	Reagent	Functional mimic	Idiotypic level
KT	KT	-	Ag
Anti-KT	Mouse mAbKT4	KTR	Ab1
Anti-anti-KT	Rat mAb K10	KT	Ab2
Anti-anti-anti-KT	Mouse polyclonal Abs	KTR	Ab3
Anti-anti-anti-anti-KT	Mouse mAb K20	KT	Ab4
Anti-anti-anti-anti-anti-KT	TK 1–4 peptides	KTR	Ab5 mimics
Anti-anti-anti-anti-anti-anti-KT	Mouse polyclonal Abs	KT	Ab6

The remarkable ability of anti-Id Abs to mimic the original Ag has been widely reported [28]–[35].

Numerous studies indicate that the mimicry by anti-Ids is functional rather than structural and not necessarily depends on amino acid sequence homologies between protein Ag and anti-Id Ab. This means that an anti-Id provides similar binding interactions rather than exact topological replicas of the Ag (or a mimicking Ab) at a molecular level. This would be particularly true when anti-anti-anti-Id Abs (such as mAb K20) mimic molecules which are not Abs (such as mAb K10) but glycoproteins of other nature (such as KT), by binding to the same area of the Ab1 (mAb KT4), or by making similar binding interactions to those made by the Ag (KT) with its own specific KTR. In this sense, these latter interactions (KT-KTR) could be similar to those between selected Ab5-like peptides and elicited Ab6, suggesting that these peptides represent functional KTR immunogenic mimics, as they stimulate the production of candidacidal Abs. Since KTR seems to consist of β-1,3-glucans [18], this study confirm the potential of peptides, as those selected from phage display libraries, to mimic carbohydrate Ags, although the molecular mechanisms that enable such mimicking remain to be elucidated.

As *C. albicans* is a pathogenic yeast, which is often responsible for human disease in the form of oral infections, vaginitis and invasive infections, the latter being particularly frequent in immunocompromised patients [36], [37], renewed interest in effective vaccines has been spurred by the emergence of resistance to and the relatively high toxicity of antifungal agents [38], [39]. No such vaccine, however is currently available. β-1,3-glucan is expressed on the fungal cell wall and is believed to represent the receptor for the KT produced by strains of *W*. *anomalus* (*P. anomala*). This toxin is interesting because of a particularly expanded spectrum of antifungal activities which may extend beyond candidal strains [40]. Coherently, experimental “universal” antifungal vaccines based on a conjugate between algal laminarin, a β-1,3-glucan molecule, and a protein carrier have been recently described [19], [41], [42]. We explored and demonstrated here the possibility of selecting peptide mimics of the KTR from different random peptide libraries. Among the mAb K20-reactive phage clones with 4 different amino acid sequences, two bore a consensus sequence, while no other discernible consensus motif could be observed. This seems to confirm the previously described ability of different peptide sequences to recognize a single mAb, thus providing more than one structural solution for binding to a given Ab combining site [24]. Taking advantage of the ease and versatility of a recently described DNA immunization approach [25], [26], we demonstrated the ability of the 4 selected gene constructs, particularly when administered in combination with plasmids containing the IFN-γ gene, and of KLH-conjugated peptides, to elicit in most of the immunized animals a candidacidal serum activity.

TK4, in particular, either as DNA construct or protein conjugate, proved to be the most immunogenic, thus confirming the different ability of peptide mimotopes to induce an immune response directed against the external Ag [24]. It should be noted, in this respect, that some of the DNA-immunized animals failed to produce detectable candidacidal responses. The use of latest techniques for DNA delivery or second-generation mimics designed on the basis of existing peptides and/or guided by detailed structural analysis of the interaction between the nominal Ag and the Ab combining site, may increase the magnitude of the candidacidal response and/or the frequency of responders [43].

Overall, our findings show that idiotypy may overcome the barrier represented by animal species (mouse/rat/mouse), nature of immunogens (toxin/antibodies/mimics) and type of immuno-technology (hybridomas/phage display/peptide/DNA immunization).
